# Correction: Peripheral mitochondrial DNA, telomere length and DNA methylation as predictors of live birth in *in vitro* fertilization cycles

**DOI:** 10.1371/journal.pone.0296603

**Published:** 2024-01-02

**Authors:** Letizia Li Piani, Marco Reschini, Edgardo Somigliana, Stefania Ferrari, Andrea Busnelli, Paola Viganò, Chiara Favero, Benedetta Albetti, Mirjam Hoxha, Valentina Bollati

There are errors in the Funding section. The correct Funding statement is: This study was partially funded by Italian Ministry of Health, Current research IRCCS.
