# Supplementary material for: Peripheral mitochondrial DNA, telomere length and DNA methylation as predictors of live birth in in vitro fertilization cycles
Source: PLoS One. 2022 Jan 24;17(1):e0261591. doi: 10.1371/journal.pone.0261591 (PMC8786209; doi:10.1371/journal.pone.0261591)
Supplement: S1 Table — (DOCX) [file pone.0261591.s001.docx]

**S1 Table.** Biomarkers of aging in women who did and did not achieve a live birth according to the IVF indication

| Unexplained infertility |  |  |  |
| --- | --- | --- | --- |
| Variable | Live birth | NO live birth | p |
|  | n=15 | n=40 |  |
|  |  |  |  |
| Mitochondrial DNA (copy number) | 0.94 ± 0.19 | 1.03 ± 0.28 | 0.25 |
| Telomere length (TTAGGG repeats) | 1.00 ± 0.15 | 0.99 ± 0.22 | 0.76 |
| LINE-1 methylation (%5mC) | 76.55 ± 1.85 | 76.64 ± 1.47 | 0.86 |
| DNAm Age (years) | 37.47 ± 3.5 | 37.25 ± 3.64 | 0.84 |
| Age acceleration (years) | 0.36 ± 3.35 | 0.31 ± 3.56 | 0.96 |
|  |  |  |  |
|  |  |  |  |
|  |  |  |  |
| Unovulatory infertility |  |  |  |
| Variable | Live birth | NO live birth | p |
|  | n=4 | n=6 |  |
|  |  |  |  |
| Mitochondrial DNA (copy number) | 0.89 ± 0.18 | 1.32 ± 0.41 | 0.09 |
| Telomere length (TTAGGG repeats) | 1.22 ± 0.25 | 1.08 ± 0.29 | 0.45 |
| LINE-1 methylation (%5mC) | 76.98 ± 1.05 | 75.87 ± 1.03 | 0.14 |
| DNAm Age (years) | 36.75 ± 3.69 | 38.33 ± 2.34 | 0.43 |
| Age acceleration (years) | 0.48 ± 2.84 | 1.72 ± 2.16 | 0.20 |
|  |  |  |  |
|  |  |  |  |
|  |  |  |  |
| Endometriosis |  |  |  |
| Variable | Live birth | NO live birth | p |
|  | n=10 | n=23 |  |
|  |  |  |  |
| Mitochondrial DNA (copy number) | 0.99 ± 0.28 | 1.07 ± 0.27 | 0.41 |
| Telomere length (TTAGGG repeats) | 1.02 ± 0.23 | 0.98 ± 0.25 | 0.66 |
| LINE-1 methylation (%5mC) | 76.05 ± 1.26 | 76.28 ± 1.42 | 0.66 |
| DNAm Age (years) | 33.50 ± 5.52 | 36.57 ± 2.41 | 0.03 |
| Age acceleration (years) | -3.35± 5.69 | -0.31 ± 2.38 | 0.04 |
|  |  |  |  |
|  |  |  |  |
|  |  |  |  |
| Tubal factor infertility |  |  |  |
| Variable | Live birth | NO live birth | p |
|  | n=8 | n=18 |  |
|  |  |  |  |
| Mitochondrial DNA (copy number) | 1.12 ± 0.25 | 1.03 ± 0.28 | 0.46 |
| Telomere length (TTAGGG repeats) | 1.06 ± 0.34 | 0.95 ± 0.18 | 0.28 |
| LINE-1 methylation (%5mC) | 76.38 ± 1.12 | 76.84 ± 1.95 | 0.54 |
| DNAm Age (years) | 37.0 ± 3.30 | 36.56 ± 4.03 | 0.79 |
| Age acceleration (years) | 0.30 ± 2.79 | -0.46 ± 4.14 | 0.64 |
|  |  |  |  |
|  |  |  |  |
|  |  |  |  |
| Male factor infertility |  |  |  |
| Variable | Live birth | NO live birth | p |
|  | n=18 | n=30 |  |
|  |  |  |  |
| Mitochondrial DNA (copy number) | 1.07 ± 0.35 | 1.04 ± 0.29 | 0.79 |
| Telomere length (TTAGGG repeats) | 1.04 ± 0.21 | 1.06 ± 0.37 | 0.84 |
| LINE-1 methylation (%5mC) | 76.59 ± 1.50 | 76.38 ± 1.61 | 0.66 |
| DNAm Age (years) | 36.22 ± 4.29 | 37.67 ± 3.26 | 0.19 |
| Age acceleration (years) | -0.33 ± 4.16 | 0.63 ± 3.11 | 0.37 |
|  |  |  |  |
|  |  |  |  |
|  |  |  |  |
| Mixed infertility |  |  |  |
| Variable | Live birth | NO live birth | p |
|  | n=3 | n=6 |  |
|  |  |  |  |
| Mitochondrial DNA (copy number) | 0.97 ± 0.15 | 0.81 ± 0.40 | 0.51 |
| Telomere length (TTAGGG repeats) | 1.01 ± 0.32 | 0.96 ± 0.33 | 0.85 |
| LINE-1 methylation (%5mC) | 75.63 ± 0.65 | 77.1 ± 1.35 | 0.13 |
| DNAm Age (years) | 34.33 ± 2.08 | 39.67 ± 2.66 | 0.02 |
| Age acceleration (years) | -1.57 ± 2.08 | 2.53 ± 2.05 | 0.03 |
|  |  |  |  |
| Data are reported as mean ± SD; p: p-value; DNAm Age: biological age | | | |
