# Supplementary material for: Peripheral mitochondrial DNA, telomere length and DNA methylation as predictors of live birth in in vitro fertilization cycles
Source: PLoS One. 2022 Jan 24;17(1):e0261591. doi: 10.1371/journal.pone.0261591 (PMC8786209; doi:10.1371/journal.pone.0261591)
Supplement: Attachment — Submitted filename: PONE-D-21-26763_Reviwer_1.docx [file pone.0261591.s003.docx]

This work aims to analyse the potential of different age-indicators (telomere length, mitochondrial DNA and DNA methylation pattern) as possible markers of success after IVF treatments.

The topic is of interest for the reproductive field. The manuscript is well-written, and authors provide key references to the whole comprehension of the work. However, there are several points that could be improved:

Major points:

-The authors recruited a total of 181 patients for IVF or ICSI. These people had different pathologies: endometriosis, anovulatory problems, tubal factor, even the male factor could be affecting the results. It is possible that the imprint of aging changes in a different manner in different pathologies. In addition, it does not seem compatible to find a common pattern between women with a specific reproductive pathology and women undergoing IVF because of male factor.

Making subgroups of patients, according to each pathology would help to understand whether the analysed biomarkers (telomere length, mitochondrial DNA and DNA methylation pattern) do work for any given pathology, rather than for all of them together. It would be better to compare the results of age markers of each subgroup/pathology with healthy controls of similar age.

-Line 232 and 233: “The contrast of our findings with those reported by Busnelli et al. is more difficult to reconcile”. Could you please try to give a possible explanation for these differences, for example referring to the limitations of either your or their study?

-Please explain the meaning of your results to facilitate the reading of the manuscript. For example: Odds ratio of 0.9 (in the case of mDNA) would indicate that the probability of having a live newborn is lower each time your DNA methylation fingerprint corresponds to a year older.

-Table 2. The number of oocytes retrieved, and the number of cleavage stage embryos is repeated twice with different values. Please explain better in the text or below the table what is the difference between both results. It is really not easy to understand.

Minor points:

-Line 65 and Line 72: “Aging” and “ageing” should be unified.

-Line 122: “sample” should be replaced by “samples”.

-Line 245: “Leucocytes” should be replaced by “Leukocytes”.

-Line 243, 247 and 249: “telomere length” should be replaced by “TL”.

-Line 202 and onwards: The type of statistical analysis performed should be explained in materials and methods, and only a brief reference could be done in results if necessary.

-Since the manuscript body refers to the DNAm age when speaking of “Biological age (years)”, please explain write underneath the table that equivalence because it is confusing.
