# Supplementary material for: Peripheral mitochondrial DNA, telomere length and DNA methylation as predictors of live birth in in vitro fertilization cycles
Source: PLoS One. 2022 Jan 24;17(1):e0261591. doi: 10.1371/journal.pone.0261591 (PMC8786209; doi:10.1371/journal.pone.0261591)
Supplement: Attachment — Submitted filename: Response to Reviewer.docx [file pone.0261591.s005.docx]

**Response to Reviewer** #1

*The short title of the paper overemphasises the results of the paper. The authors state that the association is “insufficient to claim a clinical use”. The paper is an interesting indicator for further research to understand the genetic basis of oocyte aging rather than clinical testing for treatment. Line 339: this final clause also still implies the predictive potential for DNAm. Knowing how the IVF sector responds to such comments (another expensive unhelpful test for the patient) it would be ethically more acceptable to be cautious. In practice, the predictive power would need to be many times more powerful to alter patient selection.*

We thank the Reviewer for the comment. We completely agree that DNAm age should be much more powerful to be considered useful for a clinical purpose. However, in our conclusion we wanted only to emphasize the urgency of exploring ovarian DNAm aging mechanisms, not inviting to adopt it clinically. As short title could be misleading, we modified it. Similarly, we added a sentence to clarify the message before line 339.

*Abstract/ Objective Line 27.The brackets around (biological age…rate) are confusing implying that these are the factors that are part of DNAm calculation.*

We thank the Reviewer for the comment, the sentence was modified as requested.

Line 142 equates Biological age and DNAm age so why not use just one term? The paper is written assuming that the main readers will be those in the field of Reproductive medicine/science so it is better to write to their understanding.

Corrected.

*Reviewer #2: Reviewer 2 is satisfied with the response of the authors. Some of the points raised by reviewer 2 were written under “reviewer 1”. However, reviewer 2 has checked all the comments that were raised by Reviewer 2 from the beginning.*

We would like to thank the Reviewer for the comment.
